# Complete Genome Sequences of the Xylose-Fermenting *Candida intermedia* Strains CBS 141442 and PYCC 4715

**DOI:** 10.1128/genomeA.00138-17

**Published:** 2017-04-06

**Authors:** Antonio D. Moreno, Christian Tellgren-Roth, Lucile Soler, Jacques Dainat, Lisbeth Olsson, Cecilia Geijer

**Affiliations:** a Department of Biology and Biological Engineering, Industrial Biotechnology, Chalmers University of Technology, Gothenburg, Sweden; b Department of Immunology, Genetics and Pathology, National Genomics Infrastructure, Science for Life Laboratory, Uppsala University, Uppsala, Sweden; c Department of Medical Biochemistry and Microbiology, National Bioinformatics Infrastructure Sweden, Uppsala University, Uppsala, Sweden

## Abstract

Sustainable biofuel production from lignocellulosic materials requires efficient and complete use of all abundant sugars in the biomass, including xylose. Here, we report on the *de novo* genome assemblies of two strains of the xylose-fermenting yeast *Candida intermedia*: CBS 141442 and PYCC 4715.

## GENOME ANNOUNCEMENT

For commercially viable lignocellulose-based ethanol production, the microorganism of choice must be able to ferment all monosaccharides to ethanol, including xylose (1). The yeast *Candida intermedia* is known for its capacity to grow on and ferment xylose (2–4). We have sequenced the genomes of two strains of this yeast: CBS 141442, isolated from the liquid fraction of a steam-pretreated wheat straw hydrolysate in Gothenburg, Sweden, and PYCC 4715, isolated from sewage in Oeiras, Portugal, and which has been characterized previously in terms of xylose growth and transport capacity (2, 5).

DNA was extracted as described elsewhere (6), and samples were sent for single-molecule real-time (SMRT) sequencing (Uppsala Genome Center at the National Genomics Infrastructure, SciLifeLab, Uppsala, Sweden). DNA was sheared into 10-kb fragments using a GeneMachines HydroShear instrument (Digilab, Marlborough, MA, USA). SMRT bells were constructed and sequenced on three SMRT cells on a Pacific Biosciences RSII sequencer according to the manufacturer’s instructions (Pacific Biosciences, Menlo Park, CA, USA) with a 4-h movie time.

For *de novo* assembly of the two genomes, reads were assembled using the SMRT Analysis HGAP3 assembly pipeline; 450 Mb of subreads longer than 8.3 kb and 4 kb were used for the preassembly step for the CBS 141442 and PYCC 4715 genomes, respectively, and 369 Mb of corrected reads with an average read length of 8.5 kb (CBS 141442) and 323 Mb of corrected reads with an average read length of 5.5 kb (PYCC 4715) were used to assemble the genomes with the Celera assembler included in SMRT Analysis. The assemblies were polished using Quiver (Pacific Biosciences). To assess the completeness of the assemblies, contig ends were analyzed for repetitive sequence motives. For CBS 141442, contigs were manually joined at unique overlaps to create complete chromosomes. For PYCC 4715, a reference-guided assembly with the CBS 141442 chromosomes as a backbone was used to create full-length chromosomes. Gap-filling was done using Quiver (Pacific Biosciences).

Annotations of the *C. intermedia* strains were computed using the Maker package version 2.31-8 (7). For construction of the gene models, *ab initio* predictions from three sources were combined: a profile model for *Candida guilliermondii* included with Augustus version 2.7 (8), a profile model for the SNAP gene predictor based on the annotation of *Clavispora lusitaniae* (9), and a self-trained GeneMark-ES version 4.3 *ab initio* model specific for fungi (10). To support gene predictions, a protein data set was provided (manually curated protein sequences from UniProt), and publically available expressed sequence tag data from the genome of *Candida albicans*.

The CBS 141442 and PYCC 4715 genomes each consist of seven chromosomes, totaling 13,162,108 and 13,077,109 nucleotides, respectively. In total, 5,944 (CBS 141442) and 6,082 (PYCC 4715) protein-coding genes were found. The genome sequences and the identified genes provide insights into how *C. intermedia* utilizes xylose, which can be used to improve xylose fermentation in lignocellulosic bioethanol production.

### Accession number(s).

The sequence data for *C. intermedia* strains CBS 141442 and PYCC 4715 have been deposited in the European Nucleotide Archive (ENA) with the accession numbers LT635756 to LT635763 and LT635764 to LT635771, respectively.
